# PRAK Promotes the Pathogen Clearance by Macrophage Through Regulating Autophagy and Inflammasome Activation

**DOI:** 10.3389/fimmu.2021.618561

**Published:** 2021-04-16

**Authors:** Ligu Mi, Yan Wang, Hui Xu, Yu Wang, Jia Wu, Hui Dai, Yu Zhang

**Affiliations:** ^1^ Department of Immunology, School of Basic Medical Sciences, National Health Commission (NHC) Key Laboratory of Medical Immunology, Peking University, Beijing, China; ^2^ Department of Immunology, School of Basic Medical Sciences, Shihezi University, Shihezi, Xinjiang, China; ^3^ Institute of Biological Sciences, Jinzhou Medical University, Jinzhou, China

**Keywords:** autophagy, pathogen, phagocytosis, macrophage, p38-regulated/activated protein kinase

## Abstract

The p38 regulated/activated protein kinase (PRAK) is a protein kinase downstream of p38MAPK. The present study investigated its function in the macrophage. Myeloid-specific deletion of *Prak* resulted in a significant reduction in F4/80^+^CD11b^+^ peritoneal macrophages with decreased expression of MHC-II and CD80. Upon infection with *Listeria monocytogenes*, *Prak*-deficient mice demonstrated an increased mortality, which was accompanied by a higher bacterial load in multiple tissues and elevated levels of proinflammatory cytokines in the serum. While the *Prak*-deficient macrophage showed similar potency in phagocytosis assays, its bactericidal activity was severely impaired. Moreover, *Prak* deficiency was associated with defects in ROS production, inflammasome activation as well as autophagy induction. Therefore, PRAK critically contributes to the clearance of intracellular pathogens by affecting multiple aspects of the macrophage function.

## Introduction

Phagocytosis mediated by macrophages plays an important role in the first line defense against invading pathogens. This continuous process starts from the internalization of the microorganism, leading to phagosome formation. The phagosome is then fused with hydrolytic enzyme-containing lysosomes to generate a phagolysosome. Several conditions favor bacterial elimination in the phagolysosome, including reactive oxygen species (ROS), reactive nitrogen species, lysozyme, and antibacterial inflammatory cytokines such as IFN-γ (1).

Phagocytosis and subsequent respiratory burst exhibit highly effective pathogen clearance, especially for extracellular bacterial (2). However, several intracellular bacterial pathogens have evolved multiple mechanisms to avoid or circumvent the phagocytotic activity. Alternative approaches are thus needed to eliminate intracellular bacterial. In the last decade, autophagy emerged as a critical protective mechanism employed by the host to restrict the intracellular bacterial spread (3). After the autophagy induction by starvation or rapamycin in RAW 264.7 macrophages, the *M. tuberculosis* variant Bovis BCG was shown to be colocalized with the LC3^+^ compartments. Primarily, the autophagic pathway facilitates the clearance of intracellular pathogens by enhancing phagosome-lysosome fusion (4, 5). However, autophagy-activating mechanisms that lead to the beneficial outcomes of antimicrobial responses are less clear (3).

The p38-regulated/activated protein kinase (PRAK) belongs to the MAPK-activated protein kinase family. Upon phosphorylation and activation by p38MAPK, PRAK contributes to the regulation of a range of cellular responses, including cell proliferation, migration, and stress responses. Our previous studies have demonstrated that PRAK dysfunction impaired the formation of neutrophil extracellular traps (NETs) and the antibacterial activity of neutrophils, most likely through autophagy-triggered neutrophil apoptosis (6). While there is no clear evidence for the participation of PRAK in inflammation and pathogen elimination by macrophage, PRAK transcripts were reported to be downregulated in the peripheral blood samples of patients with tuberculosis compared to healthy controls (7), pointing to its potential involvement in host responses to bacterial infection. However, *Prak*-deficient and wild type mice were equally susceptible to LPS-induced endotoxic shock. Moreover, no significant difference was observed in the biosynthesis of TNF-α, IL-6, or IFN-γ by spleen cells from wild type and *Prak*-deficient mice after LPS stimulation (8). These conflicting results suggest a complex role of PRAK in pathogen clearance.

To investigate the PRAK function in macrophage-mediated defense, *Prak* ^flox/flox^ mice were crossbred with *LysM*-Cre mice to achieve *Prak* deletion in the myeloid lineage. Phagocytosis and bacterial killing of wild type and *Prak*-deficient macrophages was compared using *in vitro* assays and the *Listeria monocytogene* (LM)-induced sepsis model. Furthermore, we analyzed the potential influence of *Prak* deficiency on the production of cytokines and ROS and the activation of inflammasome and autophagy in macrophages. Data obtained from these studies indicated an important role of PRAK in phagocytic killing and bacterial removal by macrophages.

## Materials and Methods

### Mice

*Prak* ^flox/flox^ mice were purchased from Shanghai Model Organisms Center (Shanghai, China), *LysM-*Cre mice were a gift from Prof. Xiaoyu Hu (Tsinghua University, China). Myeloid cell-specific *Prak* knockout mice (KO) were generated by crossbreeding *Prak* ^flox/flox^ mice with *LysM*-Cre mice. *LysM*-Cre-negative, *Prak* ^flox/flox^ littermates served as wild type (WT) controls. All mice were maintained in the specific-pathogen-free animal facility of Peking University Health Science Center. All animal experiments were approved by the Ethics Committee of Peking University Health Science Center (Project identification code: LA2019094).

### Macrophage Isolation and Induction

Mice were injected with 4% thioglycollate (TG) into the peritoneal cavity. After 96 h, macrophages were collected by flushing with ice cold PBS, plated and incubated at 37°C for 2 h in RPMI 1640 with 0.5% FBS. After removal of non-adherent cells by PBS washing, the adherent macrophages were used for further experiment. The purity of macrophages was >95%, as determined by flow cytometry using CD11b and F4/80 as markers.

### Generation of Bone Marrow-Derived Macrophages

Bone marrow (BM) cells are harvested from femur and tibia. After removal of erythrocytes with RBC lysis buffer (Tonbo Biosciences, San Diego, CA), BM cells were plated in RPMI-1640-containing 10% fetal bovine serum. Non-adherent cells were harvested next day and re-plated at a density of 4x10^6^/plate in petri dishes in the RPMI-1640 medium containing macrophage colony-stimulating factor (100 ng/ml, R&D Systems, Minneapolis, USA). Bone marrow-derived macrophages (BMDM) were harvested at day 7 for further experiments.

### Reagents and Antibodies

Recombinant Mouse M-CSF and IFN-γ were purchased from R&D. Lipopolysaccharide (LPS) and anti-LC3 antibody were purchased from Sigma Aldrich (St Louis, USA). 2’,7’-dichlorofluorescin diacetate (DCFDA) was from Life Technologies (Carlsbad, USA). Antibodies against NLRP3 were purchased from Cell Signaling Technology. Anti-CD11b (M1/70) and anti-F4/80 (BM8.1) were from BD-Bioscience. Alexa Fluor 488 goat anti-rabbit IgG, anti-mouse IgG and Hoechst 33342 were from Zhongshan Golden Bridge Biotechnology. Anti-NOX2 (ab129068) was purchased from Abcam.

### Cytokines/Chemokines Detection

The expression of cytokines and chemokines in the serum at day 0, 3, and 5 post-infection was measured by Luminex multifactor assay using a magnetic bead format (R&D, USA). A total of 14 cytokines and chemokines were analyzed, including IL-1α, IL-1β, IL-6, TGF-α, G-CSF, GM-CSF, IFN-γ, TNF-α, IL-10, MCP-1/CCL2, IL-12p70, IP-1α/CCL3, MIP-1β/CCL4, and CXCL10/IP-10. Assays were conducted according to the manufacturer’s recommendations. Data were collected by Luminex 200 (Luminex, Austin, TX, USA) and analyzed with Bio-plex manager software 6.0.

### Immunofluorescence Microscopy

Peritoneal macrophages were placed on glass coverslips in 24-well-plates and incubated with 200U/ml IFN-γ for 24 h at 37°C. Subsequently, cells were fixed with 4% paraformaldehyde for 2 h and permeabilized with 0.1% Triton X-100 for 5 min at RT. After incubation with blocking buffer (5 mg/ml BSA in PBS) at 37°C for 1 h, cells were stained with the primary antibody overnight at 4°C. After three washes with PBS, the cells were incubated for another 1 h with the secondary goat anti-rabbit IgG or anti-mouse IgG antibody conjugated with Alexa Fluor 488. The DNA was counterstained with Hoechst 33342 at RT for 5 min. Images were captured with confocal microscope.

### Western Blotting

Macrophages lysate was prepared with lysis buffer (50 mM Tris, pH 7.5, 1% Triton X-100, 150 mM NaCl) plus protease inhibitor cocktail (Roche, Mannheim, Germany) and fractionated on SDS-PAGE before being transferred onto polyvinylidene fluoride (PVDF) membranes. The PVDF membranes were probed by incubating with the appropriate primary antibodies at a dilution of 1:500 overnight at 4°C, followed by incubation with the horseradish peroxidase (HRP)-conjugated secondary antibody (diluted at 1:5,000) for 1 h at room temperature. The protein bands were visualized with a SuperSignal WestPico Kit according to the manufacturer’s instructions (Thermo Fisher).

### Infection With *Listeria monocytogenes*

*Listeria monocytogene* (LM, ATCC 19115) was obtained from China General Microbiological Culture Collection Center. Frozen aliquots of bacteria were inoculated into Brain-Heart-Infusion (BHI) broth and allowed to grow to OD 0.1–0.4 (1 OD equals to 2 × 10^9^ CFU/ml). The culture was harvested and resuspended in PBS. Each mouse received an intraperitoneal injection of 7.5 × 10^6^ bacteria. Animals were weighed and monitored daily for mortality for up to 14 days. To study bacterial clearance, A sub-lethal dose (5 × 10^6^ CFU) of LM* *was intraperitoneally administrated. Mice were humanely euthanized by exposure to CO_2_ and cervical dislocation at day 3 post-infection. Peritoneal lavage, peripheral blood, liver and spleen tissues were collected, and the bacterial burdens were enumerated by serial dilution of an aliquot (1-100μl) of samples on BHI agar plates.

### Assays for Phagocytosis and Bactericidal Activity

Macrophages were incubated with fluorescent microsphere (Invitrogen, F8827) at 37°C for 1 h and the phagocytosis were then stopped by adding ice-cold PBS. After three times wash with ice-cold PBS, engulfment of the beads was detected by flow cytometry. The phagocytic activity was evaluated by phagocytic percentage and phagocytic index. Phagocytic percentage was calculated as the ratio of macrophages that phagocytosed fluorescent microsphere to the number of total macrophages. Phagocytic index was calculated with the number of phagocytosed fluorescent microsphere divided by the number of macrophages that phagocytosed fluorescent microsphere. To test for phagocytosis of bacteria, *L. monocytogenes* were labeled by incubating with 5mM carboxyfluorescein succinimidyl ester (CFSE) at 37°C for 30 minutes in dark, followed by washing with PBS twice.

Antibiotic protection assay was employed to determine the phagocytic and bactericidal activities of macrophages as previously described (9). Macrophages were incubated with *L. monocytogenes* for 1 h at a MOI of 50. The phagocytic activity was measured by counting the CFU inside the macrophage immediately after three times wash with PBS [CFU (P)]. After further incubation for 4 h in the presence of 50 µg/ml gentamicin (Sigma) to kill the bacteria in the culture medium, the bacteria remaining viable inside the macrophage were again counted using colony forming assay [CFU (B)]. The bactericidal activity was calculated as follows: [1-CFU (B)/CFU (P]×100 (%).

### Quantification of ROS Generation

Intracellular ROS was determined using the fluorescent probe, 2’,7’-dichlorofluorescin diacetate (DCFDA). Briefly, macrophages were stimulated with *L. Monocytogenes* for 1 h, followed by incubation with DCFDA for 20 min. After washing with PBS, DCFDA fluorescence was detected using flow cytometry.

### Statistical Analysis

Statistical analyses were performed using SPSS 17.0 and GraphPad Prism 7. Data are presented as the mean ± standard deviation (SD). Two-tailed Student’s *t*-test or paired *t*-test was used for single comparison, and two-way ANOVA was used for multiple comparisons. Differences in survival were analyzed by log-rank test. In all tests, P < 0.05 was considered statistically significant.

## Results

### Reduction of Peritoneal Macrophages Upon Myeloid Cell-Specific Deletion of Prak

**Figure 1:**
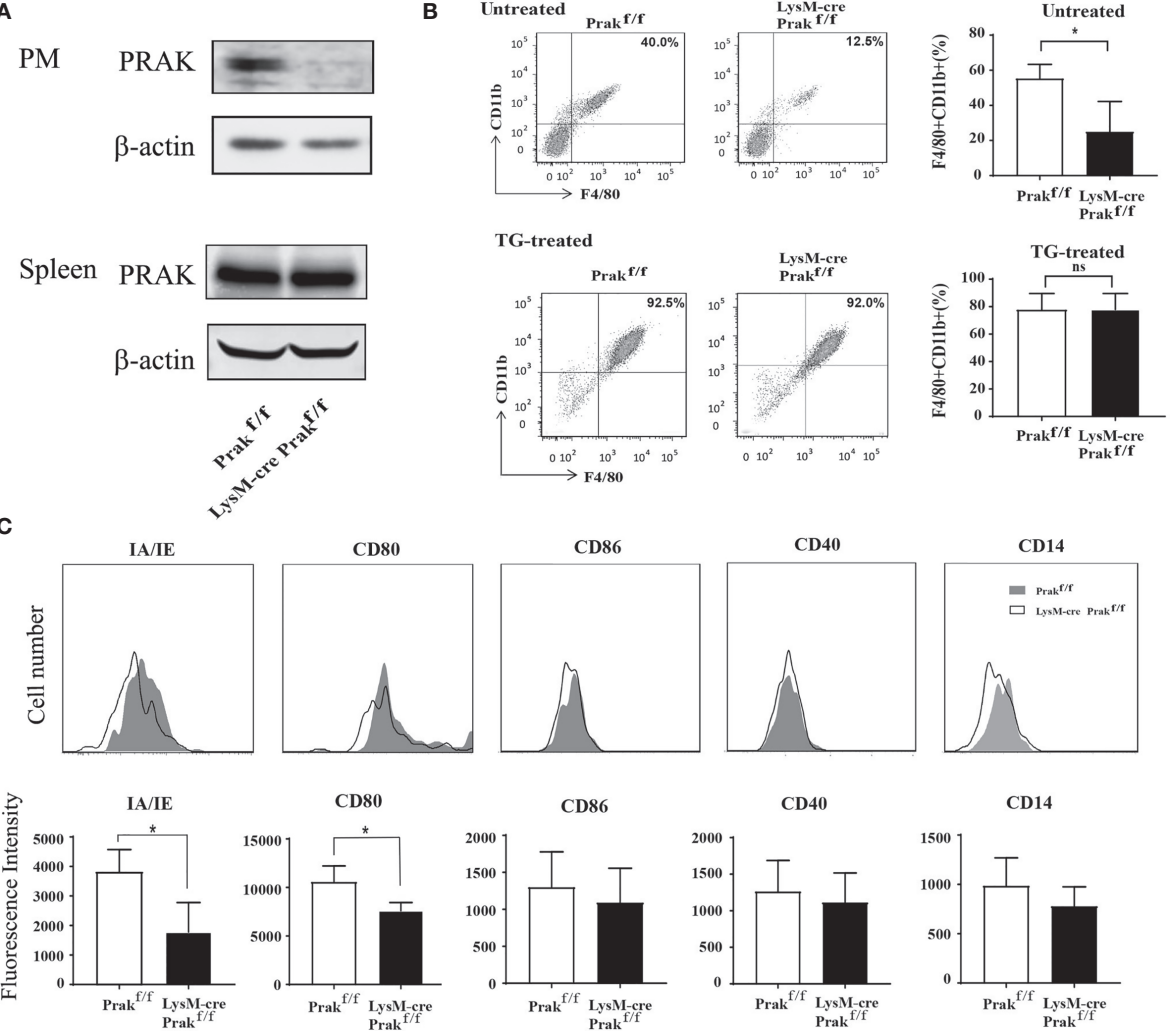
Characterization of mice with Myeloid-specific deletion of *Prak.* **(A)** Peritoneal macrophages (PM) and total splenocytes were collected from *Prak* knockout (KO, *LysM*-Cre *Prak* ^flox/flox^) and wild type (*Prak* ^flox/flox^) mice. The expression of PRAK protein was detected by Western blotting. **(B)** Total cells in the peritoneal lavage from untreated and thioglycollate (TG)-treated mice were stained for CD11b and F4/80 expression. Representative dot plots are shown on the left and the percentage of CD11b^+^ F4/80^+^ macrophages are presented as mean ± SD on the right (n=4 for each group). **(C)** Surface expression of MHC II, CD80, CD86, CD40 and CD14 in *Prak*-deficient and wild type peritoneal macrophages was analyzed by flow cytometry. Representative histograms from three independent experiments are shown in the top panel and the mean fluorescence Intensity is presented as mean ± SD at the bottom. *P<0.05 by *t*-test. ns, not significant.

To investigate the function of PRAK in the macrophage, mice with myeloid cell-specific deletion of *Prak* were generated by crossbreeding *LysM*-Cre and *Prak* ^flox/flox^ mice. Lineage specificity of *Prak* deletion was confirmed by Western blot analysis of peritoneal macrophages and total splenocytes. As shown in **Figure 1A**, no PRAK expression was detected in peritoneal macrophages from *LysM*-Cre *Prak* ^f/f^ mice, whereas *Prak*-deficient mice and the wild type littermates showed comparable levels of PRAK in the spleen.

*Prak*-deficient mice displayed no overt defect in body weight, survival and behaviors. In analysis of the peritoneal macrophage compartment, we observed a significant reduction of the CD11b^+^F4/80^+^ population, from 64.1% in wild type mice to 21.9% in the knockout mice (**Figure 1B**). As expected, stimulation with thioglycolate greatly increased the number of macrophages in the peritoneal cavity. Intriguingly, the difference was no longer detected between the knockout mice and the littermate controls upon thioglycolate treatment (**Figure 1B**). Therefore, despite its profound impact on the accumulation of resident macrophages, *Prak* deficiency seems to be dispensable for the recruitment of macrophage to the inflammatory site. The peritoneal macrophage was next characterized for the expression of a panel of maturation markers, including MHC-II (IA/IE), CD80, CD86, CD14 and CD40. *Prak*-deficient macrophages had significantly reduced expression of MHC-II (IA/IE) and CD80 (**Figure 1C**), suggesting that PRAK might be involved in the functional maturation of macrophages as well.

### Increased Susceptibility of Prak-Deficient Mice to Listeria Infection

**Figure 2:**
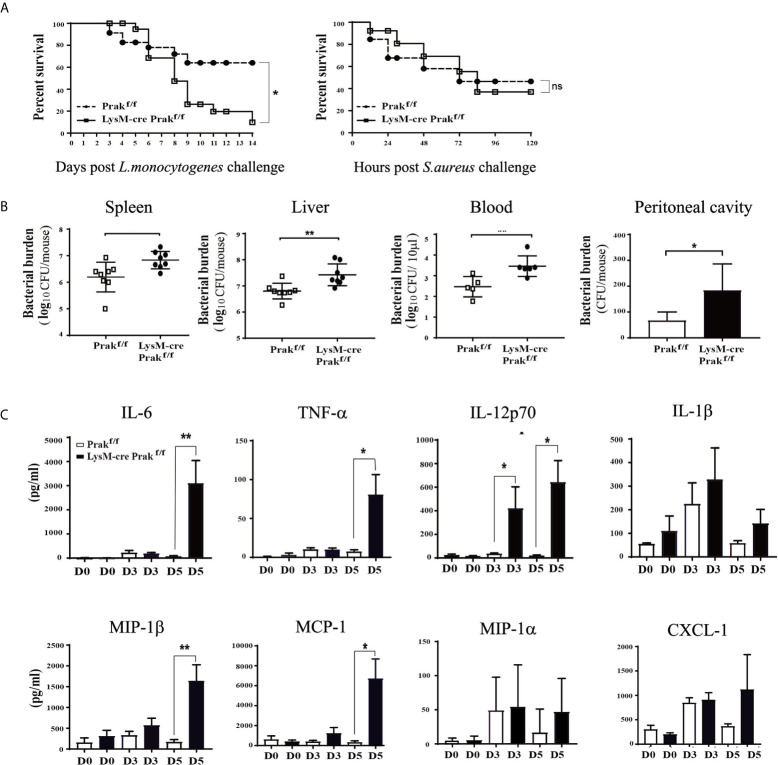
Susceptibility of Prak-deficient mice to Listeria infection. **(A)** *Prak-*deficient and wild type mice were intraperitoneally inoculated with a lethal dose of *Listeria monocytogenes* (7.5×10^6^ CFU, *Left*) or *Staphylococcus aureus* (ATCC 8325) (8×10^7^ CFU, *Right*). The survival rate was monitored daily and analyzed using the log-rank test. Each group consisted of 24 mice. *P<0.05. **(B)** Following inoculation with a sub-lethal dose (5×10^6^ CFU) of *L. monocytogenes*, the peritoneal lavage (n=3), peripheral blood (n=5), spleen (n=8) and liver tissues (n=8) were collected at day 3. Bacterial loads were measured using the colony forming assay. *P < 0.05; **P < 0.01 by *t*-test. **(C)** *Prak-*deficient (n=13) and wild type mice (n=12) received intraperitoneal injections of *L. monocytogenes* (7.5 × 10^6^ CFU). Serum samples were collected at day 0, 3 and 5, and the cytokine profile was assessed with a Luminex assay. Data from three independent experiments are presented as mean ± SD. *P < 0.05; **P < 0.01 by *t*-test. ns, not significant.

To interrogate the functional consequence of the altered macrophage compartment in *Prak*-deficient mice, we evaluated pathogen clearance by these mice following infection with intracellular or extracellular bacteria. Mice were intraperitoneally injected with *L. monocytogenes* (LM, 7.5 × 10^6^ CFUs) and monitored for survival, weight loss, and clinical signs for 14 days. In comparison to the wild type controls, *Prak*-deficient mice showed a significantly increased mortality following *L. monocytogenes* challenge (**Figure 2A**). We next compared the susceptibility to *Staphylococcus aureus*, a typical extracellular pathogen. Similar mortalities were observed in this model in the presence or absence of *Prak*. Therefore, the myeloid-specific deletion of *Prak* selectively affected the outcome of infections by intracellular versus extracellular bacteria.

To understand the mechanism underlying the elevated mortality, we assessed the efficiency of pathogen clearance following *L. monocytogene* infection. Mice received intraperitoneal injection of 5 × 10^6^ CFUs and the bacterial load was measured in the blood, spleen, liver, and peritoneal cavity at day 3 post infection. As shown in **Figure 2B**, higher bacterial loads were detected in each of tissues examined in the absence of PRAK, indicative of impaired pathogen clearance.

Cytokines are crucial mediators in the host protective responses to infectious agents. On the one hand, elevated cytokines promote immune response to eliminate pathogens. On the other hand, excessive proinflammatory cytokines destroy the balance of immune responses and induce pathological inflammatory diseases. In extreme cases, an overwhelming cytokine response called “cytokine storm” can cause tissue damage directly (10). Luminex assays were engaged to measure a panel of cytokines and chemokines in the serum at day 0, 3, 5 after *L. monocytogenes* infection. Increased levels of IL-6, TNF-α, IL-12p70, MIP-1β and MCP-1 were seen in the *Prak*-deficient mice, whereas IL-1β, MIP-1α and CXCL1 levels were comparable between the knockout and control mice (**Figure 2C**). Among them, MCP is critical for macrophage recruitment while MIP-1β drives the chemotaxis of neutrophils, macrophages and other immune cells (11).

### Impaired Killing of Engulfed Bacteria by Prak-Deficient Macrophages

**Figure 3:**
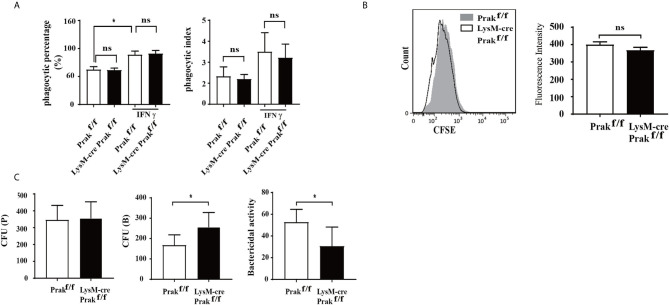
The phagocytic and bactericidal activities of *Prak*-deficient macrophages. **(A)** Peritoneal macrophages were cultured in the presence or absence of IFN-γ (200U/ml) for 24 h and then mixed with 100μl fluorescent microspheres for 1 h. Phagocytosis of the microspheres was detected by flow cytometry. The results were converted into phagocytic percentage and phagocytic index. The experiments were repeated three times. Data are presented as mean ± SD. ns, not significant. **(B)** Peritoneal macrophages from *Prak-*deficient (n=5) and wild type (n=3) mice were incubated for 1 h with CFSE-labeled *L. monocytogenes* and analyzed by flow analysis. Representative histograms from 3-5 independent experiments are shown on the left and the mean fluorescence Intensity is presented as mean ± SD on the right. ns, not statistical. **(C)** The macrophage was incubated with *L. monocytogenes* for 1 h. The number of bacteria inside the macrophage was determined immediately after washing with PBS [CFU (P)] or after further incubation for 4 h in the presence of 50 µg/ml gentamicin [CFU **(B)**]. The bactericidal activity was calculated as follows: [1-CFU (B)/CFU (P]×100 (%). Data from three independent experiments are presented as mean ± SD. *P < 0.05 by *t*-test. ns, not significant.

Phagocytosis is one of the main functions of macrophages, which is responsible for the clearance of pathogens and necrotic debris of damaged cells. To reveal any potential defect in phagocytosis associated with PRAK deficiency, we first tested the capacity of macrophages to engulf microspheres or bacteria. Resting or IFN-γ-treated macrophages were incubated with fluorescence-labeled microspheres for 1 h, followed by flow cytometric analysis. In both cases, wild type and *Prak*-deficient macrophages showed equal potency to ingest the microspheres as measured by either the percentage of phagocytic cells or the phagocytic index, which also took into consideration of the number of microsphere engulfed by individual cells (**Figure 3A**). We also assessed the phagocytosis of pathogenic bacteria. After incubation with CFSE-labeled *L. monocytogenes*, Prak-deficient and wild type macrophages displayed similar fluorescence intensity (**Figure 3B**). These results indicate that PRAK is not required for the recognition and phagocytosis of bacteria by macrophage.

The ultimate clearance of phagocytosed pathogens relies on the bactericidal action of macrophage. To assess the bactericidal activity, live *L. monocytogenes* bacteria in the infected macrophages were enumerated using colony forming assay at different time points. As shown in **Figure 3C**, similar numbers of bacteria were identified inside the wild type and *Prak*-deficient macrophages immediately after 1 h incubation with *L. monocytogenes*, which was indicative of the phagocytic activity. In contrast, more live *L. monocytogenes* were recovered from the *Prak* knockout macrophages than the wild type controls after culturing for additional 4h in the presence of gentamicin, a plasma membrane impermeable antibiotics. These data suggest that, despite the intact phagocytic activity, killing of the engulfed bacteria was impaired in the absence of PRAK.

### Suppressed ROS Production and Inflammasome Activation in Prak-Deficient Macrophages

**Figure 4:**
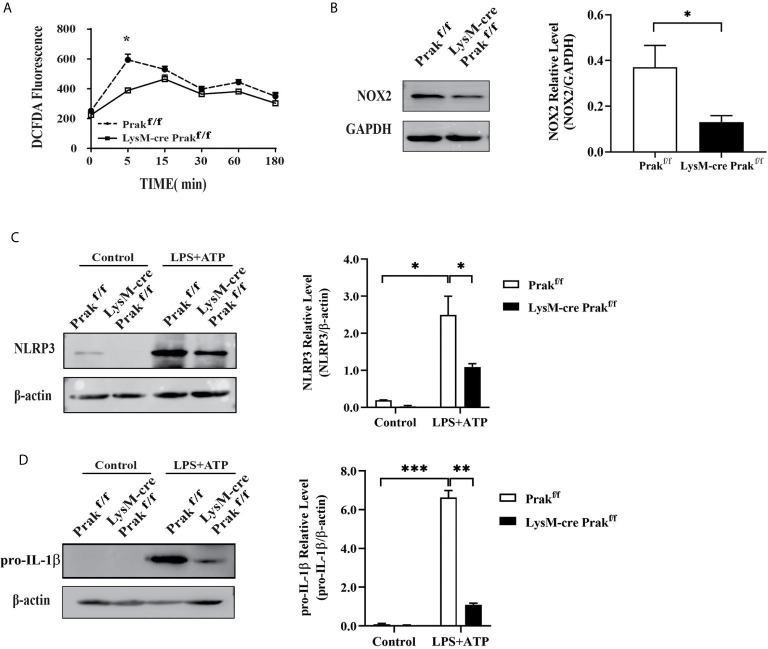
ROS production and inflammasome activation in *Prak*-deficient macrophages. **(A)** Peritoneal macrophages were incubated with *L. monocytogenes* and harvested at different time points. ROS levels were determined by staining 10 μM DCFDA for 30 min at 37°C. Data from three independent experiments are shown as mean ± SD. *P < 0.05 by two-way ANOVA. **(B)** NOX2 expression was detected by Western blotting in macrophages Representative blots out of 3 independent experiments are shown on the left. The ratio of NOX2/GAPDH is shown as mean ± SD on the right. *P < 0.05 by *t*-test. **(C, D)** TG-elicited peritoneal macrophages were allowed to rest overnight. Inflammasome activation was induced by incubating with LPS (500 ng/mL) for 4 h, followed by stimulation with ATP (3 mM) for 0.5 h. The expression of NLRP3 **(C)** and IL-1β **(D)** was assessed by western blotting. Representative blots out of 3 independent experiments are shown on the left. The ratio of NLRP3/β-actin is shown as mean ± SD on the right. *P < 0.05 by *t*-test. **P < 0.01, ***P < 0.001.

The respiratory burst, and the reactive oxygen species (ROS) produced thereby, plays a vital role in pathogen clearance by macrophages. We examined intracellular levels of ROS following *L. monocytogenes* exposure using DCFDA as a probe. In wild type cells, *L. monocytogenes* infection induced a sharp increase in ROS production within 5 min. PRAK deficiency significantly attenuated ROS production (**Figure 4A**). Consistent with the reduced ROS levels, the expression of NOX2, one of the major enzymes catalyzing the generation of ROS (12), was found to be suppressed in *Prak*-deficient cells (**Figure 4B**). ROS production is closely linked with inflammasome activation. On the one hand, ROS promotes the activation of inflammasome. On the other hand, cytokines, such as IL-1β downstream of inflammasome activation further enhances ROS production (13). A low but constitutive expression of NLRP3 was detected in resting wild type macrophages. Upon induction with LPS plus ATP, NLRP3 was seen to be dramatically increased. In the absence of PRAK, such a response was markedly attenuated (**Figure 4C**). Along with the altered NLRP3 expression, pro-IL-1β levels were found to be markedly elevated in wild type macrophages, but not as much in *Prak*-deficient ones (**Figure 4D**). We failed, however, to detect any difference in the level of mature IL-1β between wild type and *Prak*-deficient macrophage-derived cultures (data not shown). The inconsistency between pro- and cleaved-IL-1β warrants further study.

### Impaired Autophagy Induction in Prak-Deficient Macrophages

**Figure 5:**
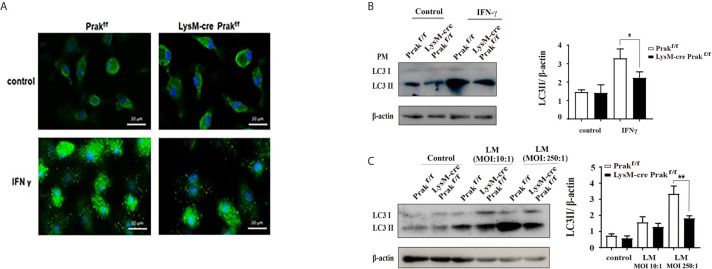
Autophagy induction in *Prak*-deficient macrophages. **(A)** TG-elicited peritoneal macrophages from *Prak-*deficient and wild type mice were allowed to rest overnight and then treated with or without IFN-γ for 24 hours. Confocal microscopy examination was performed after staining with anti-LC3 (green) and DAPI (blue). Images are representative of 3-4 independent experiments. Scale bars, 20μm. **(B)** Cells were processed as in **(A)** Western blotting was performed to detect the generation of LC3II. β-actin served as a loading control. Representative blots from 3-4 independent experiments are shown on the left. The ratio of LC3II/β-actin is shown as mean ± SD on the right. *P < 0.05 by *t*-test. **(C)** Peritoneal or bone marrow-derived macrophages were incubated *with L. monocytogenes* at a MOI of 10 or 250 for 24 h. LC3II levels were revealed by Western blotting. Representative blots from four independent experiments are shown on the left. The ratio of LC3II/β-actin is shown as mean ± SD on the right. *P < 0.01 by *t*-test; **P < 0.001 by *t*-test.

Autophagy constitutes another important mechanism for the clearance of intracellular pathogens by macrophages. To reveal whether *Prak* deletion led to altered autophagic activities, peritoneal macrophages were first treated with IFN-γ, which is known to be a potent inducer of autophagy in the macrophage (3). Immunofluorescene staining demonstrated the formation of LC3 punctum following IFN-γ treatment. Fewer puncta, however, were detected in *Prak*-deficient cells (**Figure 5A**). Autophagy induction was also monitored by Western blot analysis of LC3II. As shown in **Figure 5B**, IFN-γ treatment resulted in much elevated levels of LC3II in wild type macrophages, whereas less significant increase was observed in the knockout cells. Furthermore, we looked into autophagy induction following *L. monocytogene* infection. Increased levels of LC3II were detected in *L. monocytogene*-infected cells, which was most obvious with a MOI of 250. Again, *Prak*-deficient macrophages exhibited lower autophagic activities than the wild type cells (**Figure 5C**). Together, these results indicate that PRAK is also involved in autophagy regulation in the macrophage.

## Discussion

The present study has revealed a previously unrecognized function of PRAK in pathogen clearance by macrophages. *Prak* deficiency led to a reduction in the peritoneal macrophage compartment, along with decreased expression of maturation markers. When challenged with *L. monocytogenes*, *Prak*-deficient mice showed a higher mortality than wild type mice. Moreover, there were increased bacterial loads in multiple tissues of the knockout mice. Further analyses indicated that, while having no impact on phagocytosis, *Prak* deficiency impaired the bactericidal activity of and hence the pathogen clearance by macrophages.

Several factors may contribute to the suppressed killing associated with *Prak* deficiency, including reduction in ROS production, inflammasome activation and autophagy induction. ROS plays an essential role as an antibacterial effector and a signaling molecule in phagocytes. Pathogen exposure typically induces a rapid and sharp increase in intracellular ROS levels. Such a response was damped in *Prak*-deficient macrophages, which might be partly due to the decreased expression NOX2. NLRP3 inflammasome activation is also critical for host defense and pathogen elimination in fungal, bacterial, and viral infections (14, 15). In the presence of LPS and ATP, wild type macrophages displayed a much elevated levels of NLRP3, its expression was only seen to be modestly increased in *Prak*-deficient macrophages, indicative of impaired inflammasome activation. By targeting cytosolic constituents for lysosomal degradation, autophagy represents an important homeostatic mechanism. Evidence is accumulating that autophagy is also a major player in the elimination of intracellular pathogens by macrophages (4, 5). Numerous exogenous and endogenous signals may induce the cascade of autophagic response, including pathogen-associated molecular patterns (PAMPs), host-derived damage-associated molecular patterns (DAMPs), and cell stress (16, 17). Notably, the autophagic activities induced by either IFN-γ or *Listeria* were significantly reduced in the absence of PRAK.

As effective pathogen clearance requires the coordinated action of ROS, inflammasome and autophagy in macrophages, inhibition of any of these activities could cause a defect in the bactericidal function. However, it should be pointed out that these activities are closely interconnected. The complicated interplay encompasses both positive and negative regulatory mechanisms. The NLRP3 inflammasome can be triggered by elevated ROS levels. The inflammatory factors such as IL-1 β produced by NLRP3, on the other hand, can further enhance ROS production (18). Intracellular ROS produced by host cells may also induce bactericidal autophagy by inhibiting PI3K/Akt/mTOR signaling pathway (19). In contrast, autophagy is generally believed to be a negative regulator for inflammasome activation and ROS production (20). With respect to the inflammasome response, autophagy primarily exerts its inhibitory effect by removing inflammasome-activating endogenous stimuli or directly degrading inflammasome components including the interleukin precursors (21). Mitophagy is a special form of autophagy, which selectively eliminates excessive or damaged mitochondria to avoid the overproduction of ROS and hence the hyperactivation of inflammasome (22). Consistent with these findings, autophagy deficiency has been reported to enhance ROS generation and IL-1α release in macrophages infected by *Mycobacterium tuberculosis* (23). As such, one may anticipate that the autophagic defect in *Prak*-deficient macrophages could result in an increase in ROS production and inflammasome activation. Contrary to this expectation, we found that these activities were attenuated as well in *Prak*-deficient macrophages, highlighting the complexity in the regulation of macrophage-mediated immunity. Further studies are warranted to understand the molecular mechanisms by which PRAK regulates these diverse cellular processes.

In comparison to wild type mice, *Prak*-deficient mice showed increased levels of inflammatory cytokines and chemokines, including IL-6, TNF-α, IL-12 p70, MIP-1β and MCP-1, in the serum upon *Listeria* infection. As suggested by many previous studies (24), these proinflammatory mediators, especially TNF-α, IL-6, and IL-12 should be at least partly responsible for the increased mortality in *Listeria*-infected *Prak*-deficient mice. As far as the mechanism is concerned, we speculate that it is most likely due to the impaired pathogen clearance. The increased bacterial load continuously stimulates the cytokine production by the host immune system. Alternatively, the increased cytokine secretion may be partly attributable to the defect in autophagy. Production of cytokines has been shown to be regulated by autophagy (25–27). Mice with *Atg5* deficiency in myeloid cells have a higher bacillary load, increased inflammation and IL-1α or IL-17 production in response to *M. tuberculosis* infection than their autophagy-proficient littermates (28). Moreover, Atg16 L1-deficient macrophages displayed enhanced NLRP3 inflammasome activation and IL-1β and IL-18 production when stimulated with PAMP (29, 30).

It is worth mentioning that *LysM-*Cre also results in *Prak* deletion in neutrophils. In fact, we have previously demonstrated that PRAK is required for neutrophil-mediated defense by facilitating the generation of neutrophil extracellular traps (6). Therefore, there is a concern about the cell specificity of PRAK action in the model used in the present study.

Despite the role of macrophages in host defense against intracellular bacteria is well established (31–33), several studies have shown that other myeloid cells especially neutrophils are also critically involved in the control of *Listeria* infection (34–37), particularly in liver infection (38) and in the early phase of infection (39). As such, although our *in vitro* studies with purified macrophages clearly showed a regulatory role of PRAK in macrophage response to *Listeria* challenge, it is difficult, at this point, to distinguish the relative contribution of defects in macrophages versus neutrophils to the increased mortality of *Listeria*-infected *Prak*-deficient mice.

In summary, this study demonstrates that PRAK plays an important role in the clearance of intracellular pathogens by macrophage. More specifically, PRAK enhances the bactericidal acidity by promoting ROS production, inflammasome activation and autophagy induction. The role of PRAK in oxidative stress, inflammasome and macrophage autophagy provides a new perspective for innate immune against bacteria.

## Data Availability Statement

The original contributions presented in the study are included in the article/supplementary material. Further inquiries can be directed to the corresponding authors.

## Ethics Statement

The animal study was reviewed and approved by Ethics Committee of Peking University Health Science Center.

## Author Contributions

Study conception and design: HD and YZ. Mouse model collection: LM. Acquisition of data: LM, YaW, JW and HX. Analysis and interpretation of data: HD and YuW. All authors contributed to the article and approved the submitted version.

## Funding

This work was supported by grants from the National Natural Science Foundation of China (31970840; 81972041), Beijing Natural Science Foundation (7172112) and Non-profit Central Research Institute Fund of Chinese Academy of Medical Sciences (2019PT320006).

## Conflict of Interest

The authors declare that the research was conducted in the absence of any commercial or financial relationships that could be construed as a potential conflict of interest.

## Abbreviations

BMDM, Bone Marrow-derived Macrophages; DAMP, Damage-associated molecular pattern; DCFDA, 2’,7’-dichlorofluorescin diacetate; IFNγ, Interferon γ; IL-6, Interleukin 6; LM, *Listeria monocytogene*; LC3, microtubule-associated protein 1 light chain 3; MAPK, mitogen-activated protein kinase; MCP-1, monocyte chemotactic protein 1; M-CSF, Macrophage Colony Stimulating Factor; MIP-1α, macrophage inflammatory protein 1α; MOI, multiplicity of infection; NOX, NADPH oxidase; PAMP, pathogen-associated molecular patterns; PM, peritoneal macrophages; PRAK/MK5, p38-regulated/activated protein kinase/MAPK-activated protein kinase 5; ROS, reactive oxygen species; TNFα, Tumor necrosis factor α.
